# A Hierarchical Multimetal Oxides@Graphene Fabric Electrode with High Energy Density and Robust Cycling Performance for Flexible Supercapacitors

**DOI:** 10.1021/acs.nanolett.5c00104

**Published:** 2025-03-06

**Authors:** Yunchuan Liu, Yongzhe Zhang, Chao Yang, Muhammad Wakil Shahzad, Yichen Yan, Lixin Dai, Wangyang Lu, Wenxing Chen, Ximin He, Ben Bin Xu, Guan Wu

**Affiliations:** † National Engineering Lab for Textile Fiber Materials and Processing Technology, Zhejiang Sci-Tech University, Hangzhou 310018, P. R. China; ‡ Zhejiang Provincial Innovation Centre of Advanced Textile Technology, Shaoxing 312000, P. R. China; § Department of Mechanical and Construction Engineering, Northumbria University, Newcastle Upon Tyne NE1 8ST, U.K.; ∥ Department of Materials Science and Engineering, University of California, Los Angeles (UCLA), Los Angeles, California 90095, United States

**Keywords:** multimetal oxides, wearable applications, flexible supercapacitor, metal oxides@graphene fibers

## Abstract

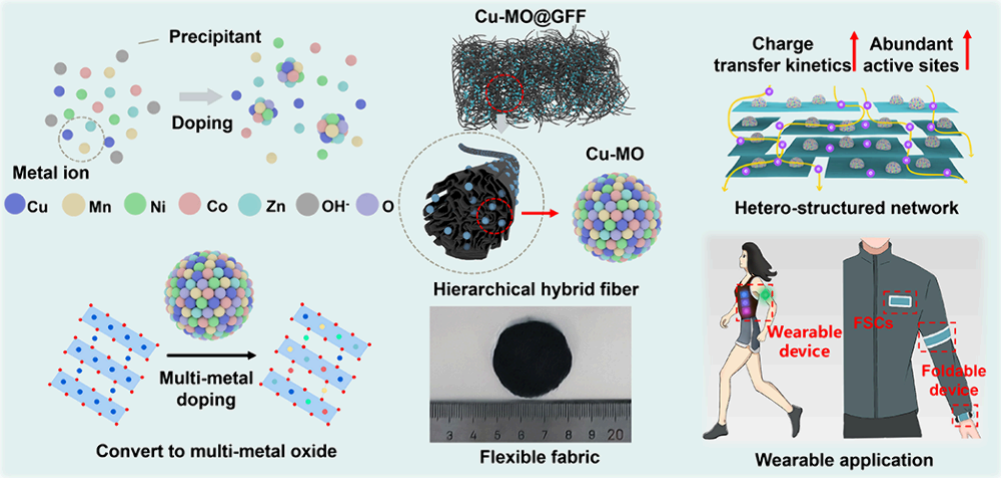

An advanced structure capable of hosting large electrochemical activity with desired balance in ion diffusion kinetics, faradic charge storage, and robust stability is the key to developing high-performance fabric-based electrochemical supercapacitors (FSCs). Herein, we develop a hierarchical multimetal oxides@graphene fabric (Cu-MO@GFF) as a supercapacitor electrode with accelerated ionic diffusion, adsorption energy, faradic redox reaction kinetics, and electrochemical reversibility. As a result, the Cu-MO@GFF presents excellent mass capacitance (534 F g^–1^), high rate performance (266 F g^–1^ at 10 A g^–1^), and good cycle performance (96.9% capacitive retention after 20,000 cycles) in 6 mol L^–1^ (M) KOH electrolyte. In addition, the Cu-MO@GFF-based solid-state FSC delivers excellent energy density (11.875 Wh kg^–1^), much-improved cyclic stability, and bending capability. On account of the excellent electrochemical behavior, this solid-state FSC can flexibly power various wearable devices (such as luminous tags, bracelets, and wearable watches), which will offer a new avenue for innovating next-generation wearable energy devices.

The quest for an innovative power solution is ongoing for modern electronics, along with their rapid developments in wearable electronics, artificial intelligence, and smart skins.^1−6^ A fiber supercapacitor (FSC) appears to be a promising candidate for its excellent flexibility, light weight, super cyclic stability, excellent rate performance, and high power density.^7−11^ While research on electrode materials, e.g., carbon-based materials, transition metal compounds, etc., has been extensively performed,^12−15^ the FSC still suffers from lower ionic/electronic conductivity and poor reversible redox activities, resulting in a low energy density and unfavored cycling performance.^16−20^ A novel electrode material that can fulfill the FSC with a desired pseudocapacitive reaction and structural stability remains yet to be exploited.

Various conductive carbonaceous nanomaterials (i.e., MXene, graphene), hold great promise in FSCs due to their remarkable electrical conductivity, large specific surface area, and superior mechanical properties.^21−23^ Zhou et al. reported a point-line-sheet heterostructure of nitrogen-doped carbon dots/Ti_3_C_2_T_*x*_/silk nanofibers to endow the assembled solid-state FSC with a high energy density (57.9 mWh cm^–3^) and a long life cycle (82.3% capacitance retention after 40,000 cycles).^24^ Nevertheless, there is an improvement in capacity through the discontinuous porous channels and limited exposed active sites.^25−27^ To alleviate the above dilemma, microstructural design (i.e., porous/core–shell structure, hollow structure, etc.) has been proposed to adjust the porous networks and boost the active sites of charge transfer to improve energy-storage depth.^28,29^ Besides, the high-energy-density FSC can be facilitated by overcoming the intrinsically low Faradaic redox reactions.

Transition metal oxides (TMO), a group of pseudocapacitive electrode materials, can improve the charge-storage ability for supercapacitors through shifting variable transition metal valences to contribute redox reactions.^30,31^ Li et al. reported an amorphous vanadium oxide with metallic properties in asymmetric supercapacitors. The obtained H-VO_*x*_-500/CC anode delivered a high specific capacitance of 554 mF·cm^–2^ with a 97.3% capacitance retention after 5000 cycles.^32^ However, the TMO has drawn concerns about their application for sluggish reaction kinetics and poor structural stability.

To solve this puzzle, the conductive carbonaceous nanomaterials have been utilized to enhance the ionic diffusion and charge transfer kinetics of TMOs.^33^ Liu et al. constructed a planar micro supercapacitor with double metal oxide modified carbon nanotubes (WO_2_/MoO_2_@P, N-CNTs) as electrodes, which showed a high capacity of 1237 C g^–1^. The CNT accelerated the reaction kinetics through ion diffusion and charge transfer of bimetallic oxides.^34^ While the kinetics issue was addressed, the challenges of structural instability and limited charge storage depth of TMO remain to be tackled. Since the number of active sites are responsible for charge storage and the magnitude of lattice stress during the electrochemical process,^35,36^ the optimization of crystal structure^37^ holds potential to realize FSCs with high energy density. To date, there are limited reports on balancing the high energy storage and cycling behavior by adjusting the crystal structure.

Here, we develop a high-energy-density composite FSC by integrating multi metal oxides with graphene fiber-assembled fabric (Cu-MO@GFF). Based on the synergistic effects of multiple metal elements, abundant oxygen vacancies are exposed on the surface of Cu-MO@GFF, leading to accelerated ionic diffusion, adsorption energy, faradic redox reaction kinetics, and electrochemical reversibility. The obtained Cu-MO@GFF presents an excellent specific mass capacitance and high rate performance. The Cu-MO@GFF-based solid-state FSC also delivers excellent energy density, good cycle performance, and bending capability, which powers various wearable devices.

A metal doping strategy has been widely applied to cope with the sluggish reaction kinetics at electrode–electrolyte interfaces and poor stability.^38^ For the high theoretical capacity (677 mAh g^–1^), CuO is selected as a doping target together with multiple transition metal atoms (Mn, Co, Ni, and Zn). Based on the elemental similarity principle, i.e., similar radius and electronegativity of doping elements to copper, multidoped metal oxide (Cu-MO) and reduced graphene oxide fibers assembled fabric (GFF) are expected to achieve the Cu-MO@GFF.

In Figure 1a, the diagram schematically illustrates the preparation of Cu-MO@GFF composite fibers through a microfluidic process, where the GO nanosheets are oriented to the horizontal flow direction in the tapered channel. Then the obtained anisotropic GO solidifies and assembles into ordered gel fibers after injecting ethanol. Next, the dried GFF is completely reduced to form reduced graphene oxide (RGO) followed by a hydrothermal reaction of the GFF fabric and metal precursor solution. A homogeneous distribution of polymetallic elements throughout GFF can be realized by introducing various metal elements to substitute Cu elements in copper sites to increase the chaos degree significantly (Figure 1b), and the macroscopical size of the Cu-MO@GFF fabric can be seen from the photograph. Introducing various metal elements to substitute Cu elements in copper sites increases the chaos degree significantly. Consequently, the Cu-MO particle evades the layer stacking to maximally uncover the reaction sites, while the modification of polymetallic elements via optimization of the crystalline structure facilitates the electronic transfer and ionic diffusion. Such a material design improves specific capacity, considerable rate performance, and stable cycling at the same time.

**Figure 1:**
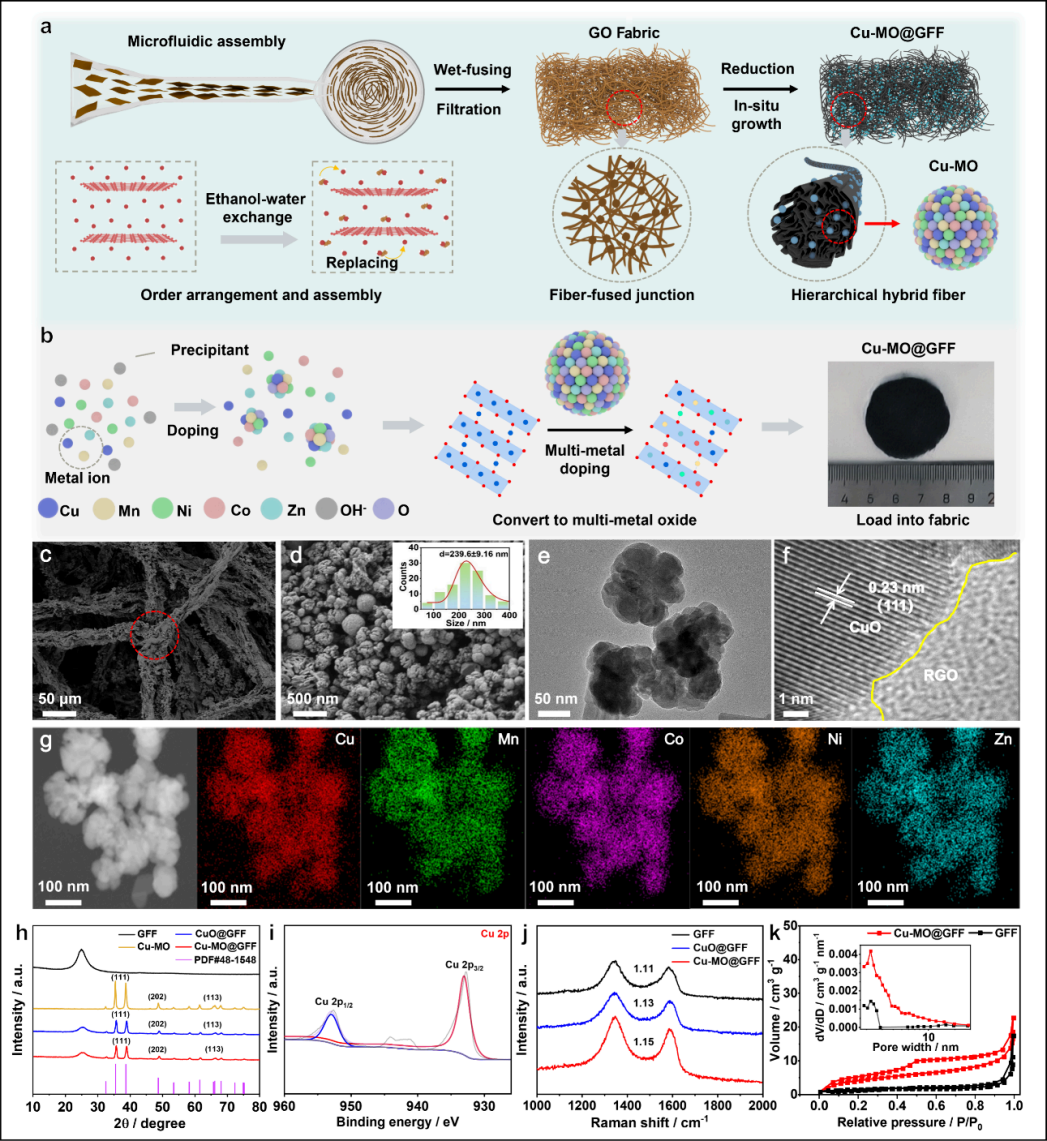
Schematic illustration for preparing Cu-MO@GFF and structure characterizations. (a) Schematic illustration for preparing Cu-MO@GFF. At this stage, the self-assembly of macroscopic GO fibers is driven by the rapid exchange of ethanol and water in the coagulation bath. After scalable manufacturing, filtration, and drying, the GFF fabrics can be achieved due to capillary forces and hydrogen bonding interactions, with an interconnected framework facilitated to offer a mechanical flexibility. (b) Self-assembly of Cu-MO by a homogeneous precipitation method and the actual picture of Cu-MO@GFF. The scale below functions as a reference. (c) SEM images of Cu-MO@GFF. (d) Image of Cu-MO particles on the surface of the RGO fiber. (e) TEM images of Cu-MO. (f) Lattice fringe pattern in Cu-MO@GFF. (g) EDS mapping of Cu, Co, Mn, Ni, and Zn elements in Cu-MO@GFF. (h) XRD patterns of GFF, Cu-MO, CuO@GFF, and Cu-MO@GFF. (i) XPS spectrum of Cu-MO@GFF for Cu 2p. (j) Raman spectra of GFF, CuO@GFF, and Cu-MO@GFF. (k) Adsorption and desorption curves and pore size distributions of GFF and Cu-MO@GFF.

As shown in Figure S1a, fibrous nanowires are uniformly distributed in the GFF, forming an interconnected network with a wrinkled and layered structure (Figure S1b). According to the SEM image of CuO@GFF shown in Figure S1d, the average diameter of the fiber is 20 ± 0.2 μm. After hydrothermal reaction, the Cu-MO nanoparticles grow uniformly on GFF fibers (Figure 1c) to reach an average size of 239.6 nm (size distribution of 50–400 nm; Figures 1d and S2). The SEM images confer a layered structure in Cu-MO@GFF. This hierarchical structure enhances the pathways for ion diffusion, fostering extensive active sites that promote thorough Faraday pseudocapacitance reaction to enable an efficient electrochemical process.

The TEM images (Figure 1e) exhibit a size distribution of Cu-MO particles of approximately 100–200 nm in the fibers, in good agreement with SEM observations. The nanoscale anchoring structure of Cu-MO on GFF is evidenced at the distinct grain boundaries between RGO and CuO (Figure 1f), where the dark phase reveals 0.23 nm lattice fringes corresponding to the (1 1 1) plane of CuO. Figure 1g exhibits the homogeneous dispersion of doping metal elements (Cu, Mn, Ni, Co, and Zn) within Cu-MO@GFF. Figure S3a shows the inductively coupled plasma–atomic emission (ICP) spectrometry of Cu-MO@GFF, showing the main element of Cu and doped elements of Mn, Co, Ni, and Zn. The total mass ratio of metal elements agrees well with the results of the TG curve in Figure S3b. In addition, the XRD patterns in Figure 1h affirm the integration of Cu-MO and GFF without the introduction of impurities, where Cu-MO@GFF and CuO@GFF exhibit concurrent characteristic peaks of GFF and Cu-MO, devoid of any extra peaks.

The electronic state of the metal element is unveiled through X-ray photoelectron spectroscopy in Figure 1i. For the Cu 2p spectrum, the peaks located at 953.23/933.45 eV correspond to Cu^2+^, indicating that the copper element in Cu-MO@GFF stably exists in the form of +2 valence. The Raman spectra in Figure 1j displays two peaks of graphene located at 1349.52 and 1584.36 cm^–1^ in GFF, CuO@GFF, and Cu-MO@GFF. The Cu-MO@GFF fiber delivers a higher *I*_D_/*I*_G_ value of 1.15 than those of the GFF (1.11) and CuO@GFF (1.13) fibers, which indicates that a high ratio of defects is introduced into the Cu-MO@GFF fiber from the multimetal doping as well as the coupling structure of metal oxides with graphene. Figures 1k and S4 depict N_2_ absorption/desorption curves and the pore size distribution of GFF and Cu-MO@GFF. The Cu-MO@GFF possesses a larger pore volume of 0.024 cm^3^ g^–1^ with a smaller average pore diameter of 7.08 nm compared with GFF (pore volume, 0.012 cm^3^ g^–1^; average pore diameter, 9.67 nm). Also, Cu-MO@GFF has a higher specific surface area of 13.25 m^2^ g^–1^, surpassing that of GFF (4.82 m^2^ g^–1^). The stress–strain curves in Figure S5 exhibit similar rupture stresses for both GFF and Cu-MO@GFF, indicating no negative impact on the mechanical strength of fabric after doping.

We next assessed the potential of GFF, CuO@GFF, and Cu-MO@GFF as supercapacitor materials by using a three-electrode setup with the adoption of 6 M KOH as the electrolyte. In Figure 2a and Figure S6a, the cyclic voltammetry (CV) curve of GFF displays a characteristic square-like shape, indicating its primary reliance on electric double layer capacitance. In striking comparison, CuO@GFF and Cu-MO@GFF exhibit obvious redox peaks at 0.2–0.4 and 0.6–0.8 V, respectively. The CV areas of CuO@GFF and Cu-MO@GFF are significantly larger than those of GFF, implying more pseudocapacitive capacity and superior energy storage capability after introducing metal oxides/Cu-MO compositions (Figures 2a and S6b and S6c). The Cu-MO@GFF has more explicit redox peaks and a larger electrochemical area, indicating an improved redox degree and charge storage.

**Figure 2:**
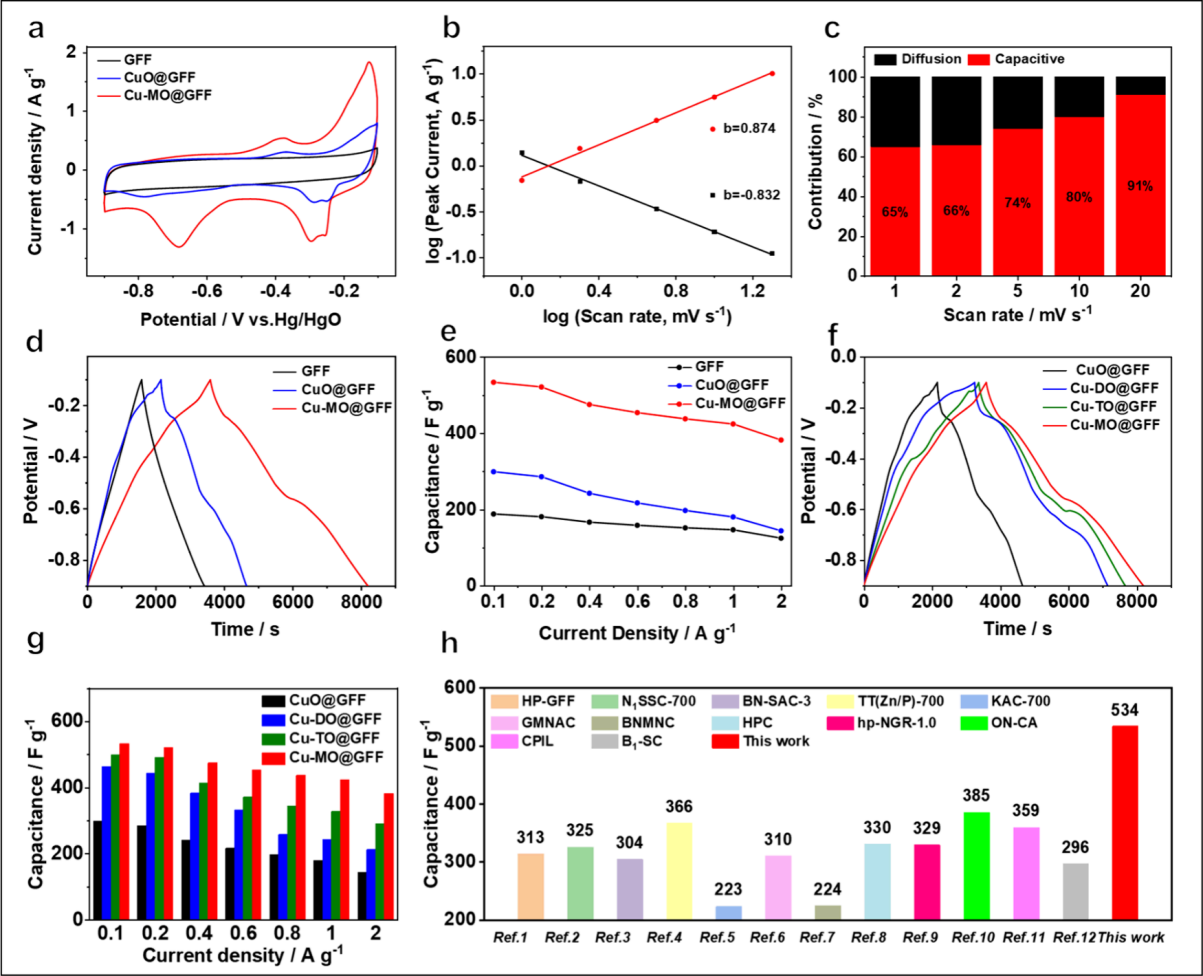
Electrochemical performance in 6 M KOH aqueous electrolyte. (a) CV curves of GFF, CuO@GFF, and Cu-MO@GFF at 1 mV s^–1^. (b) The square root of peak current density vs square root of the scan rate for Cu-MO@GFF. (c) The capacitive contribution to total stored charge of Cu-MO@GFF under various scan rates. (d) GCD curves of GFF, CuO@GFF, and Cu-MO@GFF at 0.1 A g^–1^. (e) Specific mass capacitances of GFF, CuO@GFF, and Cu-MO@GFF at different current densities, ranging from 0.1 to 2 A g^–1^. (f) GCD curves of CuO@GFF, Cu-DO@GFF, Cu-TO@GFF, and Cu-MO@GFF at 0.1 A g^–1^. Cu-DO@GFF, double-metal-doping oxide composite restored GO fiber assembled fabric; Cu-TO@GFF, triple-metal-doping oxide composite restored GO fiber assembled fabric. (g) Specific mass capacitances of CuO@GFF, Cu-DO@GFF, Cu-TO@GFF, and Cu-MO@GFF at different current densities. (h) Specific capacitance of Cu-MO@GFF with other materials.

To understand the charge storage process, the kinetic behavior of Cu-MO@GFF is analyzed (Figure 2b). According to the relationship between CV scan rate and peak current density (*I*_p_ = *av*^*b*^), the *b*-values of the reduction peak are 0.84 and 0.88 for the oxidation peak. These *b*-values mean that Cu-MO@GFF has diffusion-controlled ion insertion behavior and surface-controlled pseudocapacitive behavior. After calculating the contribution of capacitive behavior of Cu-MO@GFF to the total stored charge (Figure 2c and Figure S7),^39^ we found that the capacitive contribution increases from 65% to 91% as the scan rate increases. These data prove a good coupling of pseudocapacitive behavior from polymetallic oxides with the electronic conduction of graphene fibers.

Figure 2d illustrates the galvanostatic charge–discharge (GCD) profiles of synthesized materials. The GCD curve for GFF presents a typical symmetrical triangle curve without a charge and discharge plateau, whereas explicit plateaus appear in the CuO@GFF and Cu-MO@GFF curves. This contrast can be ascribed to the redox reactions of metal ions to offer stronger faradic charge storage capability. Then, we calculated the specific capacitance to assess the rate performances of CuO@GFF and Cu-MO@GFF (Figure 2e), according to the GCD curves (Figure S8). Evidently, Cu-MO@GFF achieves a superior specific capacitance of 534 F g^–1^ at 0.1 A g^–1^ compared to those of GFF (197 F g^–1^) and CuO@GFF (302 F g^–1^), thereby demonstrating its excellent capacity and rate performance.

Figure 2f and Figure S9 describe the galvanostatic charge/discharge (GCD) profiles of CuO@GFF, Cu-DO@GFF, Cu-TO@GFF, and Cu-MO@GFF. The charge–discharge plateaus observed for each material are attributed to the redox reactions of metal ions, which is in accordance with the results of CV curves in Figure S10. Among them, the Cu-MO@GFF electrode displays the longest charging–discharging times, which shows its stronger Faradaic charge storage. Based on the GCD curves, we calculated the specific mass capacitances of CuO@GFF, Cu-DO@GFF, Cu-TO@GFF, and Cu-MO@GFF in Figure 2g. The Cu-MO@GFF presents a higher capacitance than those of CuO@GFF, Cu-DO@GFF (464 F g^–1^), and Cu-TO@GFF (500.3 F g^–1^). Even at the rate of 2 A g^–1^, the HE-MO@GF retains the highest specific capacitance of 382.5 F g^–1^ among these fiber-based materials. Additionally, the capacitance of Cu-MO@GFF outperforms that of fiber-based electrodes recently reported in the literature (Figure 2h and Table S1).^40−51^ The above-mentioned rate performance, specific capacity, and cycling life prove the excellent electrochemical behaviors of Cu-MO@GFF, stemming from the composite structure to optimization of kinetics.

To evaluate the durability of Cu-MO@GFF, we prolonged the cycles to an impressive 20,000 at 10 A g^–1^, retaining a high retention of 96.9% with a Coulombic efficiency near at ∼100%, highlighting its exceptional cycling stability (Figures 3a and S11). After long-term cycling, the Cu-MO@GFF electrode presents a similar fiber-shape morphology and the same crystal structure of the CuO phase as the pristine electrode, which is evidenced by the SEM images and XRD patterns (Figures 3a and 3b). To state the effect of the element doping strategy on structural stability, the XRD curves and lattice parameters of Cu-MO@GFF at different charge and discharge voltages were collected (Figures 3c and 3d and Figures S12 and S13). The characteristic peak of CuO shows a negligible shift at each voltage without the change in peak intensity and the lattice parameters are reversible after cycles, indicating the high stability of the Cu-MO crystal structure along with the charging–discharging process.

**Figure 3:**
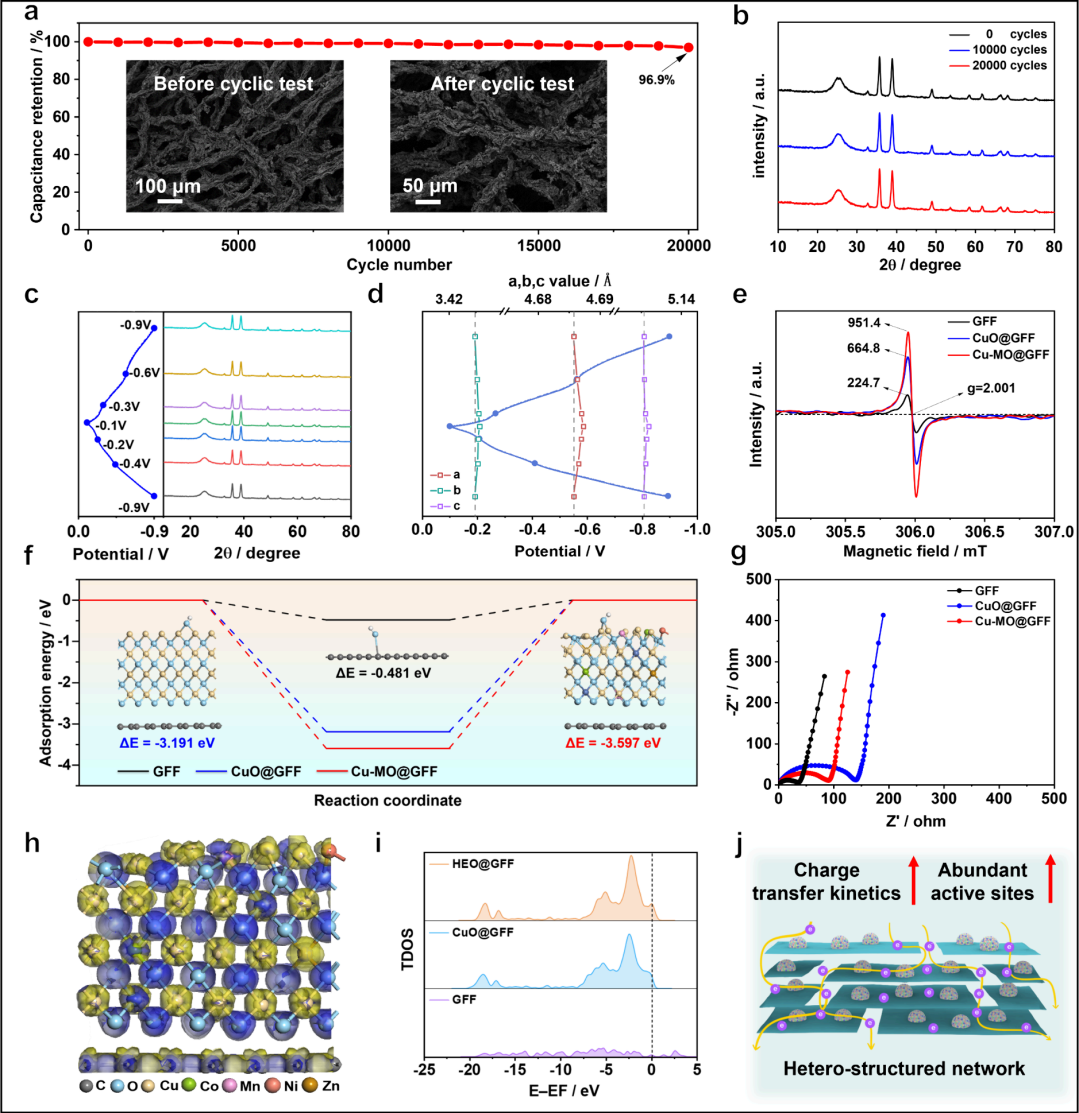
Experimental and theoretical study of energy storage mechanisms. (a) Cycling performance of Cu-MO@GFF at 10 A g^–1^. Inset: SEM images of Cu-MO@GFF before and after cycling. (b) XRD patterns of Cu-MO@GFF after 10,000 and 20,000 cycles. (c) Rietveld refinement patterns of XRD data for Cu-MO@GFF at different voltages. (d) The crystal cell parameters obtained from (b). The lengths of the *a*-, *b*-, and *c*-axes slightly enlarge as applied voltage increases in the charging process, and opposite during discharge, demonstrating a slight microstress during the electrochemical process. (e) Paramagnetic electron resonance spectra of GFF, CuO@GFF, and Cu-MO@GFF. (f) Adsorption energies of carbon sites at GFF and copper sites of CuO@GFF and Cu-MO@GFF. (g) Impedance curves of GFF, CuO@GFF, and Cu-MO@GFF in a three-electrode 6 M KOH system. (h) The differential charge of CuO@GFF and Cu-MO@GFF. (i) Total state density plots of GFF, CuO@GFF, and Cu-MO@GFF. (j) Schematic illustration for heterostructured network of Cu-MO@GFF.

Additionally, electron paramagnetic resonance spectra were recorded to reveal the oxygen vacancies (Figure 3e). The sharp peaks with a *g*-value of 2.001 are ascribed to the generation of oxygen vacancies, and the Cu-MO@GFF fiber with a highest peak intensity indicates the introduction of a high concentration of oxygen vacancies. Figure 3f compares the OH^–^ adsorption energy of various fibers with an obvious difference. GFF shows a low OH^–^ adsorption energy of −0.481 eV while CuO@GFF owns strong ion adsorption capacity toward OH^–^ (−3.191 eV), which probably comes from the limited adsorption sites to adsorb free OH^–^ in GFF compared with that in CuO. After the modification with multimetal oxides, the adsorption energy at the Cu site further increases to −3.597 eV, stating more effective adsorption sites owing to the multidoping strategy, which favors the capture of hydroxyl ions to boost the electrochemical reactions.

The electronic conductivity, electrochemical impedance spectra, and the relevant fitting results are shown in Figures 3g,S14, and S15 and Table S3. It demonstrates that multiple dopings of transition metal elements would optimize the electron conductivity of oxide to enhance the electron conductivity of the composite fiber (see Figures S14 and S15 for more discussion). The generation and dissipation of charges around transition metal atoms (Cu, Co, Ni, Mn, and Zn) in Cu-MO@GFF obviously differ from CuO@GFF (Figure 3h and Figure S16). Prior to introducing the Cu-MO, the CuO@GFF only shows less charge generation and dissipation in this composite structure. A strong enhancement in the generation and dissipation of charges in the metal oxides is discovered in Cu-MO@GFF. More charges gathered near the doped metal atoms indicate that more charges participate in the reactions, effectively stimulating the redox activity to contribute more capacity. In addition, density of states (DOS) calculations are performed to illustrate the band structures of materials (Figure 3i). A slight increase in the total DOS (TDOS) at the Fermi level is observed after the introduction of multimetal oxides in GFF. For the Cu-MO@GFF material, a distinct increase in the TDOS at the Fermi level indicates the increased probability of electrons transitioning to the conduction band, resulting in the Cu-MO@GFF material featuring metallic properties, which can be evidenced by the alternating current impedance spectra in Figure 3g. Therefore, this heterostructured network achieves faster charge transfer kinetics and abundant active sites (Figure 3j). The multimetal oxides bring abundant structural defects as well as oxygen vacancies into the crystal structure, leading to a fast ion adsorption, a high TDOS at the Fermi level, and structural stability to facilitate the charge transfer kinetics, to enable an improved energy storage capability as well as working life.

We assembled a flexible supercapacitor (FSC) based on the Cu-MO@GFF. As shown in Figures 4a and S17, the CV curves of rectangular-shaped GFF, CuO@GFF, and Cu-MO@GFF exhibit an ascending trend. In Figure 4b, the Cu-MO@GFF FSC demonstrates the highest specific capacitance of 237.5 F g^–1^, surpassing those of both GFF (132.7 F g^–1^) and CuO@GFF (101.6 F g^–1^). Notably, the Cu-MO@GFF FSC maintains a high specific capacity of 138.5 F g^–1^ at a high current density of 5 A g^–1^, indicating its exceptional energy storage capability and rate behavior (Figure 4c and Figure S18). Additionally, Figure 4d underscores the remarkable cycling durability of Cu-MO@GFF FSC, as it retains 89.3% of its capacity even after an extensive 10,000 cycles, which is superior to CuO@GFF (50.6%), much closer to the cycling performance of GFF (Figure S19). In the meantime, the impedance of Cu-MO@GFF also maintains excellent stability during the cycle (Figure S20 and Table S4). To evaluate the adaptability and wearability of FSC devices, Figure 4e illustrates its successful bending resilience across various angles, from 30–180°, demonstrating its flexibility. It is noteworthy that CV curves of Cu-MO@GFF FSC at different bending angles present no discernible capacitance degradation (Figure S21), demonstrating the excellent ability of Cu-MO@GFF FSC for bearing bending. The long-term operational tests under dynamic bending are conducted to validate its practicality. The CV curves of Cu-MO@GFF FSC after bending multiple times at 60° are shown in Figure S22. Obviously, Cu-MO@GFF FSC can still maintain high electrochemical stability after 5000 bending times at 60°, showing its excellent mechanical properties. Furthermore, the energy density of Cu-MO@GFF FSC spans from 11.88 to 6.93 Wh kg^1–^ while its power density falls from 45.48 to 356.14 W kg^–1^, which is higher than reported FSCs (Figure 4f and Table S2).^52−58^

**Figure 4:**
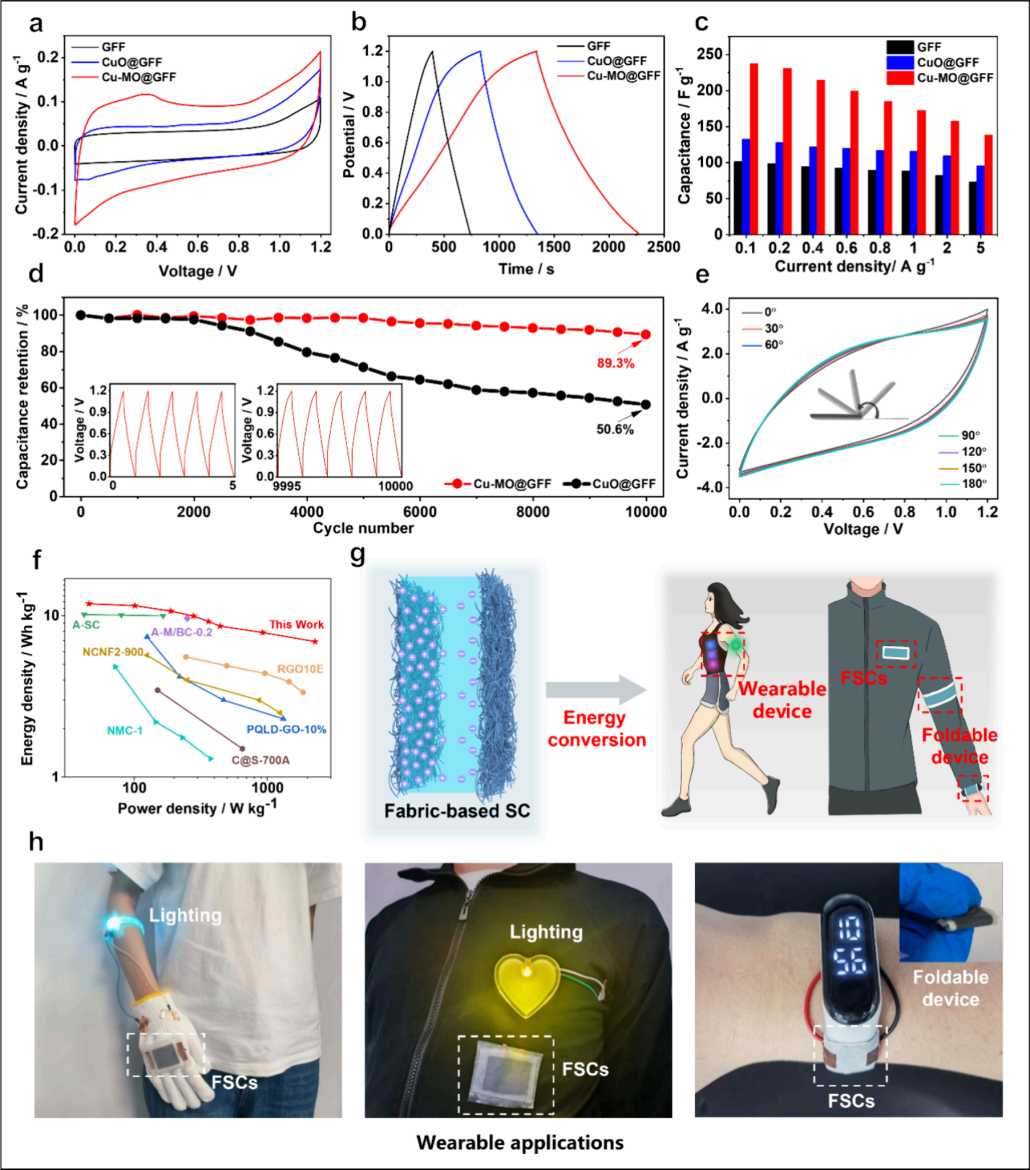
Electrochemical performances of solid-sate FSC. (a) CV curves of GFF, CuO@GFF, and Cu-MO@GFF FSC at a scan rate of 1 mV s^–1^. (b) GCD curves of GFF, CuO@GFF, and Cu-MO@GFF FSC at 0.1 A g^–1^. (c) Specific mass capacitances of GFF, CuO@GFF, and Cu-MO@GFF FSC at different current densities. (d) Cycling performance of CuO@GFF and Cu-MO@GFF FSC at 10 A g^–1^. (e) CV curves of Cu-MO@GFF FSC under the bending angles. (f) Comparison of Ragone plots of Cu-MO@GFF FSC with other fiber-based supercapacitors. (g) Schematic illustration for assembled FSC and its application characteristics. (h) Schematic diagram showing the potential application of wearable devices and the Cu-MO@GFF FSC for providing power to wearable devices.

In contrast to conventional rigid coin-type devices, fiber-based FSCs boast benefits such as seamless integration, low internal impedance, superior mechanical strength, flexibility, and convenient installation. As shown in Figure 4g, the assembled FSC can be easily woven into clothing as an energy storage device due to its excellent flexibility and foldability. On this basis, large-sized fabrics were fabricated to be integrated into smaller devices to power them (Figure S23). Besides, we tested the tolerance of Cu-MO@GFF FSC toward folding (Figure S24). Clearly, the Cu-MO@GFF FSC device can still maintain stable electrochemical behaviors after several folding times, which can guarantee that it works as a continuous and stable power supply for the electronic devices. The stress–strain curves of Cu-MO@GFF and Cu-MO@GFF FSC have proven its high mechanical stability (see Figures S25 and S26 for more discussion). Capitalizing on its excellent electrochemical and mechanical properties, we employed this Cu-MO@GFF FSC for energy storage across various diverse electronic gadgets and an electronic watch (Supplementary Movies 1, 2, 3, and 4). As shown in Figure 4h, the Cu-MO@GFF FSC demonstrates stable operation in powering LED lights. Moreover, it can serve as an energy source for wearable electronics integrated into bendable and foldable fabrics, such as illuminating bracelets or electronic watches, etc., where a consistent and reliable power supply ensures broad adoption in wearable electronic devices. Consequently, Cu-MO@GFF FSC exhibits great promise for practical implementation in cutting-edge flexible energy storage technologies.

In summary, we described a Cu-MO@GFF approach through microfluidic spinning technology and a hydrothermal reaction. The Cu-MO@GFF has a large specific surface area and a uniformly distributed graphene conductive network, while the multimetal oxide particles possess high lattice stability and improved electrochemical activity. Therefore, the Cu-MO@GFF exhibits excellent capacitance and rate capability for a supercapacitor electrode. Additionally, Cu-MO@GFF is further processed into solid-state FSC, showing a high energy density, excellent capacitance, and stable deformation ability. On this basis, FSC can be used in wearable fabrics to power various devices, which highlights the functionally advanced fabrics and substantial progress in the portable/wearable industry.

## Supporting Information Available

Experimental Section; surface and cross-sectional SEM images of GFF (Figure S1); zoomed in graph of particle size distribution and pore size distributions (Figures S2 and S4); thermal redistribution and element distribution (Figure S3); stress–strain curve of GFF and Cu-MO@GFF (Figure S5); CV curves of GFF, CuO@GFF, and Cu-MO@GFF (Figure S6); capacitive contribution of Cu-MO@GFF (Figure S7); GCD curves of GFF, CuO@GFF, and Cu-MO@GFF (Figure S8); GCD curves of Cu-DO@GFF and Cu-TO@GFF (Figure S9); CV curves of Cu-DO@GFF and Cu-TO@GFF (Figure S10); the selected charge and discharge curves of Cu-MO@GFF from cycling test (Figure S11); changes in the *a*, *b*, and *c* values of Cu-MO@GFF at various charge and discharge voltages (Figure S12); Rietveld refinement analysis of XRD data of Cu-MO@GFF (Figure S13); EIS measurement of CuO@GF and Cu-MO@GFF (Figure S14); electronic conductivity of GFF, CuO@GFF, and Cu-MO@GFF (Figure S15); the differential charge of CuO@GFF (Figure S16); CV curves of FSCs (Figure S17); GCD curves of FSCs (Figure S18); cycling performance of GFF (Figure S19); kinetic analysis of the Cu-MO@GFF impedance (Figure S20); capacitance retention of Cu-MO@GFF FSC at different bending angles (Figure S21); CV curves of Cu-MO@GFF FSC after multiple bendings at 60° (Figure S22); large-scale fabrication of Cu-MO@GFF (Figure S23); CV curves of Cu-MO@GFF FSC before and after once and twice foldings (Figure S24); stress–strain curve of Cu-MO@GFF before and after cycling (Figure S25); and stress–strain curve of Cu-MO@GFF FSC (Figure S26) (PDF)
Cu-MO@GFF FSC used to power a yellow light (MP4)
Cu-MO@GFF FSC used to power a blue light (MP4)
Cu-MO@GFF FSC used to power an electronic watch (MP4)
Cu-MO@GFF FSC used to power an illuminating bracelet (MP4)
