# Supplementary material for: A Hierarchical Multimetal Oxides@Graphene Fabric Electrode with High Energy Density and Robust Cycling Performance for Flexible Supercapacitors
Source: Nano Lett. 2025 Mar 6;25(11):4485–93. doi: 10.1021/acs.nanolett.5c00104 (PMC11926955; doi:10.1021/acs.nanolett.5c00104)
Supplement: Supplementary file 1 — nl5c00104_si_001.pdf [file nl5c00104_si_001.pdf]

Supporting Information

for

A hierarchical multi-metal oxides@graphene fabric electrode with high energy density and robust cycling performance for flexible supercapacitors

Yunchuan Liu[1,2], Yongzhe Zhang[1,2], Chao Yang[1,2], Muhammad Wakil Shahzad[3], Yichen Yan[4], Lixin Dai[4], Wangyang Lu[1,2], Wenxing Chen[1,2], Ximin He[4,*], Ben Bin Xu[3,*], Guan Wu[1,2,*]

[1] National Engineering Lab for Textile Fiber Materials and Processing Technology, Zhejiang Sci-Tech University, Hangzhou, 310018 P. R. China.

[2] Zhejiang Provincial Innovation Centre of Advanced Textile Technology, Shaoxing, 312000 P. R. China.

[3] Department of Mechanical and Construction Engineering, Northumbria University, Newcastle Upon Tyne, NE1 8ST UK.

[4] Department of Materials Science and Engineering, University of California, Los Angeles (UCLA), Los Angeles, CA 90095, USA.

Corresponding author: Dr. Ximin He Email: ximinhe@ucla.edu

Dr. Ben Bin Xu Email: ben.xu@northumbria.ac.uk

Dr. Guan Wu Email: gwu2016@zstu.edu.cn

Experimental Sections:

Materials: Flake graphite (325 mesh) with uniform particle size, potassium permanganate ($KMnO_4$, 99.5%, AR), sodium nitrate ($NaNO_3$, 99.5%, AR), sulfuric acid (H_2SO_4, 98%, AR), ethanol (CH_3CH_2OH, AR), hydrogen peroxide aqueous solution (30%, AR), copper nitrate hexahydrate(99%, AR), zinc nitrate hexahydrate (99%, AR), cobalt nitrate hexahydrate(99%, AR), nickel nitrate hexahydrate (99%, AR), and manganese nitrate solutions (50 ωt%, AR) was purchased from Shanghai Aladdin Chemical Reagent Company. Hydroiodic acid (HI, 47 ωt%, AR) was purchased from Shanghai McLean Biochemical Company. All chemicals were used without further purification.

Preparation of GFF: Graphene oxide (GO) was synthesized using a modified Hummers chemical exfoliation method. We used 12 g L^{-1} GO solution and added ammonium hydroxide to adjust the pH of the solution to 8. Then, the GO solution is injected into the microchannel, solidified with ethanol to form gel fibers, and then collected into a rotating container of ethanol solution. After microfluidic spinning, the prepared GO fibers was filtered and dried at 25°C for 3 hours to obtain a GO fibers assembled fabric. Then, the GO gel fibers assembled fabric was reduced with HI solution at 60°C for 12 hours. The sample was then washed with ethanol and deionized water and freeze-dried to obtain Restored GO fibers assembled fabric (GFF).

Preparation of CuO@GFF: Dissolve copper nitrate hexahydrate and urea in deionized water to prepare 50 mL of a mixed solution with a metal salt concentration of 0.5 mol L^{-1} and a urea concentration of 1 mol L^{-1}.Then the GFF was immersed in the precursor

solution and reacted in the Teflon-lined stainless-steel autoclave at 180°C for 12 hours. The composite fibric was then taken out, washed, and dried. Finally, the composite fibric was calcined at 220°C for 2 hours in a tube furnace to obtain copper oxide composite restored GO fibers assembled fabric (CuO@GFF).

Preparation of Cu-DO@GFF: Dissolve copper nitrate hexahydrate, cobalt nitrate hexahydrate and nickel hexahydrate in deionized water in a molar ratio of 8: 1: 1, and then urea was added thereto to prepare 50 mL of the mixed solution with a polymetallic salts total concentration of 0.5 mol· L^{-1} and a urea concentration of 1 mol· L^{-1}. Then the GFF was immersed in the precursor solution and reacted in the Teflon-lined stainless-steel autoclave at 180°C for 12 hours. The composite fibric was then taken out, washed, and dried. Finally, the composite fibric was calcined at 220°C for 2 hours in a tube furnace to obtain double-metal-doping oxide composite restored GO fibers assembled fabric (Cu-DO@GFF).

Preparation of Cu-TO@GFF: Dissolve copper nitrate hexahydrate, cobalt nitrate hexahydrate, nickel hexahydrate, and manganese nitrate solution in deionized water in a molar ratio of 12: 1: 1: 1, and then urea was added thereto to prepare 50 mL of the mixed solution with a polymetallic salts total concentration of 0.5 mol· L^{-1} and a urea concentration of 1 mol· L^{-1}. Then the GFF was immersed in the precursor solution and reacted in the Teflon -lined stainless-steel autoclave at 180°C for 12 hours. The composite fibric was then taken out, washed, and dried. Finally, the composite fibric

was calcined at 220°C for 2 hours in a tube furnace to obtain triple-metal-doping oxide composite restored GO fibers assembled fabric (Cu-TO@GFF).

Preparation of Cu-MO@GFF: Dissolve copper nitrate hexahydrate, zinc nitrate hexahydrate, cobalt nitrate hexahydrate, nickel hexahydrate, and manganese nitrate solution in deionized water in a molar ratio of 16: 1: 1: 1: 1, and then urea was added thereto to prepare 50 mL of the mixed solution with a polymetallic salts total concentration of 0.5 mol· L^{-1} and a urea concentration of 1 mol· L^{-1}. Then the GFF was immersed in the precursor solution and reacted in the Teflon -lined stainless-steel autoclave at 180°C for 12 hours. The composite fibric was then taken out, washed, and dried. Finally, the composite fibric was calcined at 220°C for 2 hours in a tube furnace to obtain multi-metal oxide composite restored GO fibers assembled fabric (Cu-MO@GFF).

Construction of FSC: The supercapacitor device was assembled with Cu-MO@GFF materials as both cathode and anode flexible electrodes, polyvinyl alcohol (PVA)/KOH gel as electrolyte and encapsulated with polyethylene terephthalate (PET) film. Preparation of PVA/KOH gel: 4 g PVA was dissolved in 30 mL deionized water and stirred at 80° C for 1h; Meanwhile, 2.4 g KOH was dissolved in 15 mL deionized water and stirred for 1h; Subsequently, the two solutions were mixed for 2 h to form homogeneous dispersion. The solid-state HE-MO@GFF FSCs were obtained by further drying under 50° C. The Flexible FSCs are fixed to the surface of electronic devices through the adhesives to power the devices.

Characterization: SEM images were taken on a Hitachi S4800 field-emission SEM system. Transmission electron microscopy (TEM) images were collected on a transmission electron microscope (Tecnai G2 F20 S-TWIN). XPS was performed using a PHI 5000C ESCA system operated at 14.0 kV. XRD was carried out using a D8-Advance Bruker AXS diffractometer (Cu Kα radiation, k = 1.5418 Å), operating at 40 kV and 40 mA. The tensile-stress curves were taken on KJ1065A with dumb-bell shaped fabrics. A multimeter was applied to measure the conductivity of fabric. Typically, a 3 × 0.2 cm (100 μm thick) fabric was used as the test sample.

Electrochemical Measurements: The electrochemical measurements were performed at room temperature using SC28-CHI660E. The three-electrode system: It is composed of Platinum plate as the counter electrode, Hg/HgO electrode as the reference electrode, GFF, CuO@GFF and Cu-MO@GFF materials as working electrode, and 6 M KOH aqueous solution as the electrolyte. The corresponding mass for GFF, CuO@GFF and Cu-MO@GFF electrode is about 0.5 mg. The specific capacitance from the constant current charge and discharge curve (GCD) is calculated based on the formula as follows:

$$C_G = \frac{I\Delta t}{G\Delta U} \quad (1)$$

$$C_A = \frac{I\Delta t}{A\Delta U} \quad (2)$$

Among them, CG (C_A), I, Δt, G(A) and ΔU are the specific mass capacitance of the electrode, discharge current, discharge time, the mass of the electrode, and the potential window during the discharge process, respectively. For the two-electrode

system on FSC devices, the calculation formulas of mass specific capacitance (C_A), energy density (E_A) and power density (P_A) are as follows:

$$C_A = 4 \times \frac{I\Delta t}{A\Delta U} \tag{3}$$

$$E_A = \frac{1}{2} \times C_A V^2 \tag{4}$$

$$P_A = \frac{3600EA}{t} \tag{5}$$

The C_A is the specific mass capacitance. I is the discharge current. A is the weight of electrode. ΔU is the potential window (U) during the discharge process.

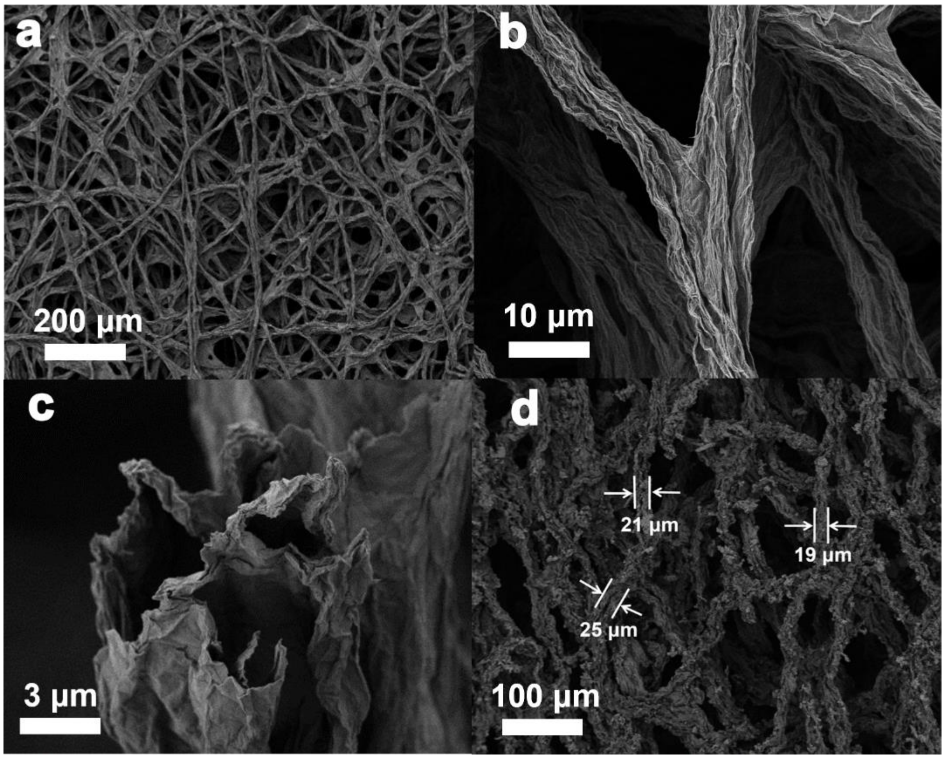

Figure S1. *(a), (b) Surface and (c) cross-sectional SEM images of GFF. (d) Surface SEM image of CuO@GFF.*

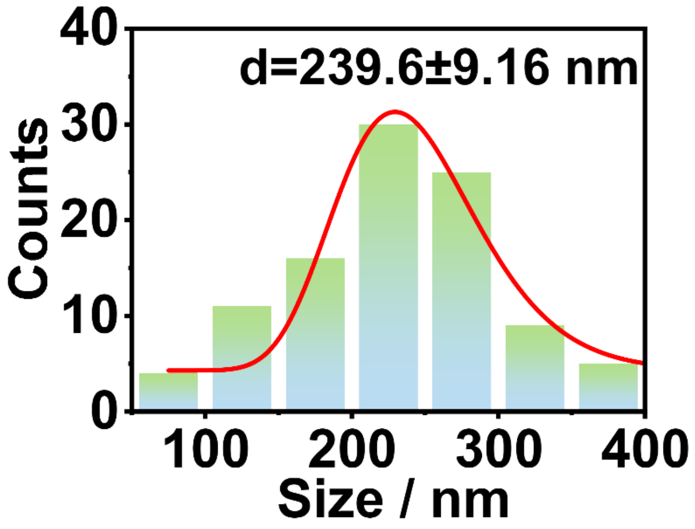

Figure S2. *Particle size distribution of Cu-MO.*

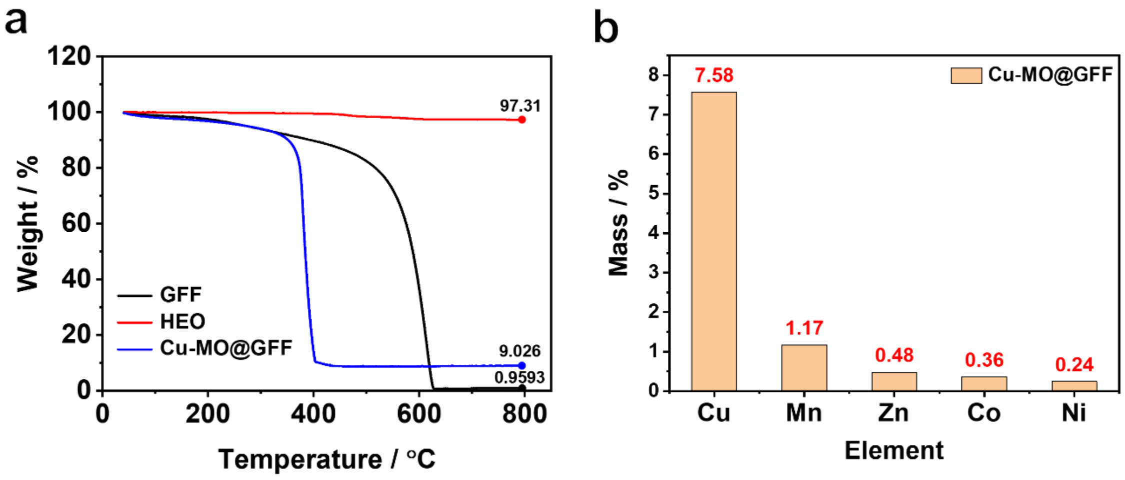

Figure S3. *(a) Thermal redistribution curves of GFF, Cu-MO and Cu-MO@GFF. (b) ICP metal element distribution of Cu-MO@GFF.*

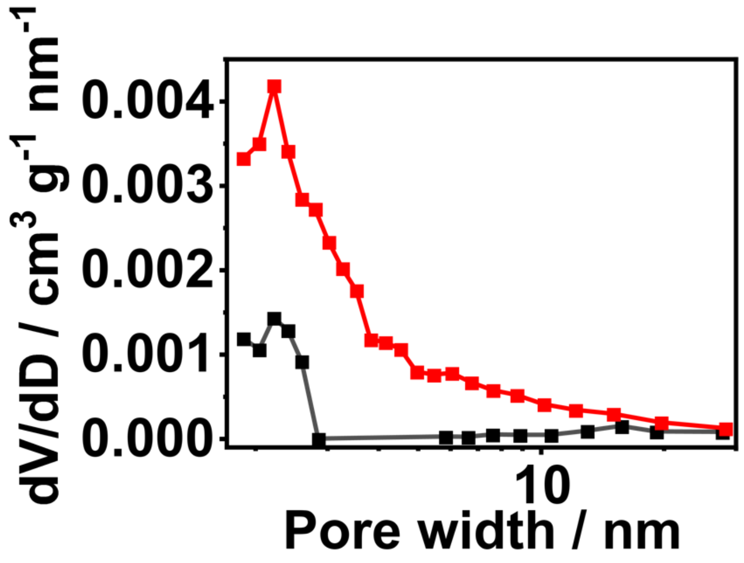

Figure S4. *Pore size distributions of GFF and Cu-MO@GFF.*

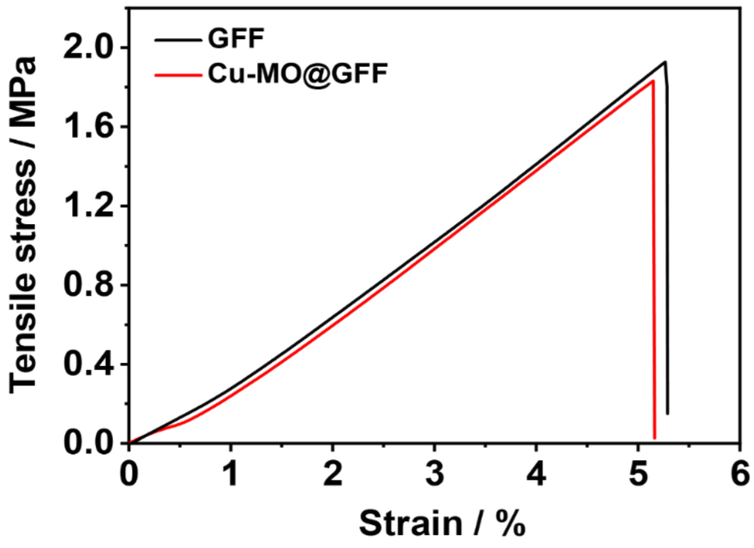

Figure S5. *Stress-strain curve of GFF and Cu-MO@GFF.*

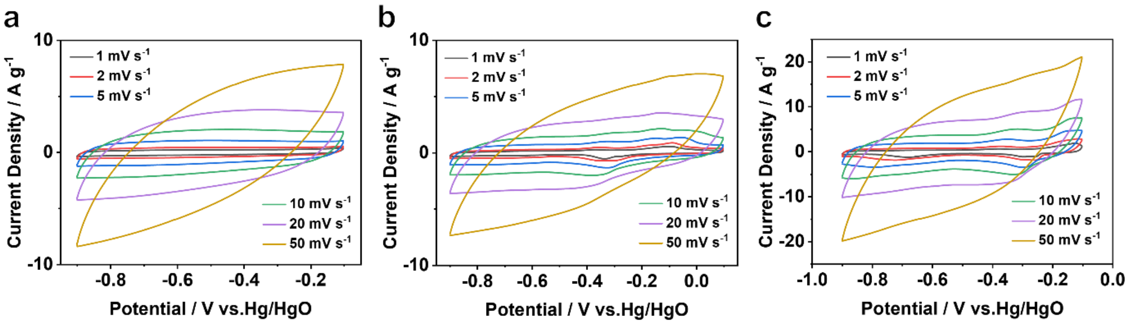

Figure S6. *CV curves of (a) GFF, (b) CuO@GFF and (c) Cu-MO@GFF at a scan rate of 1-50 mV s*$^{-1}$*.*

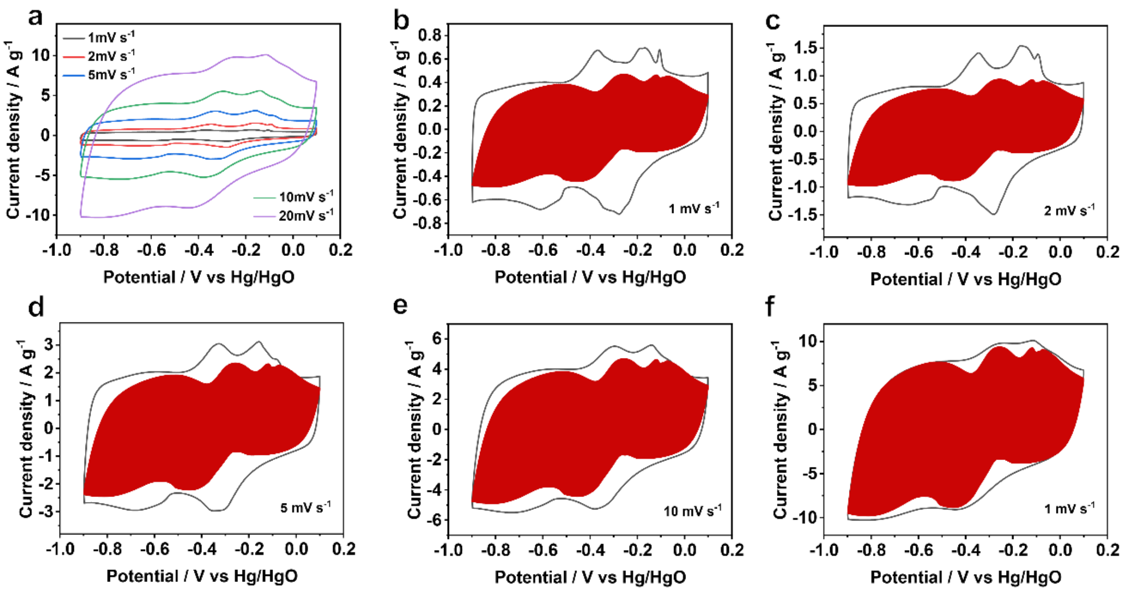

Figure S7. *(a) CV curves of Cu-MO@GFF at various scan rate and the curves at (b) 1 mV s*$^{-1}$*, (c) 2 mV s*$^{-1}$*, (d) 5 mV s*$^{-1}$*, (e) 10 mV s*$^{-1}$ *and (f)20 mV s*$^{-1}$ *respectively, where shaded area is capacitive contribution.*

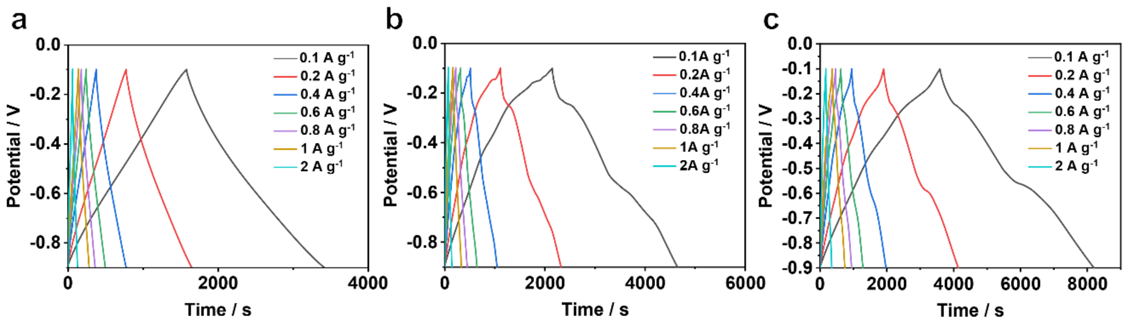

Figure S8. *GCD curves of (a) GFF, (b) CuO@GFF and (c) Cu-MO@GFF at 0.1-2 A g^{-1}.*

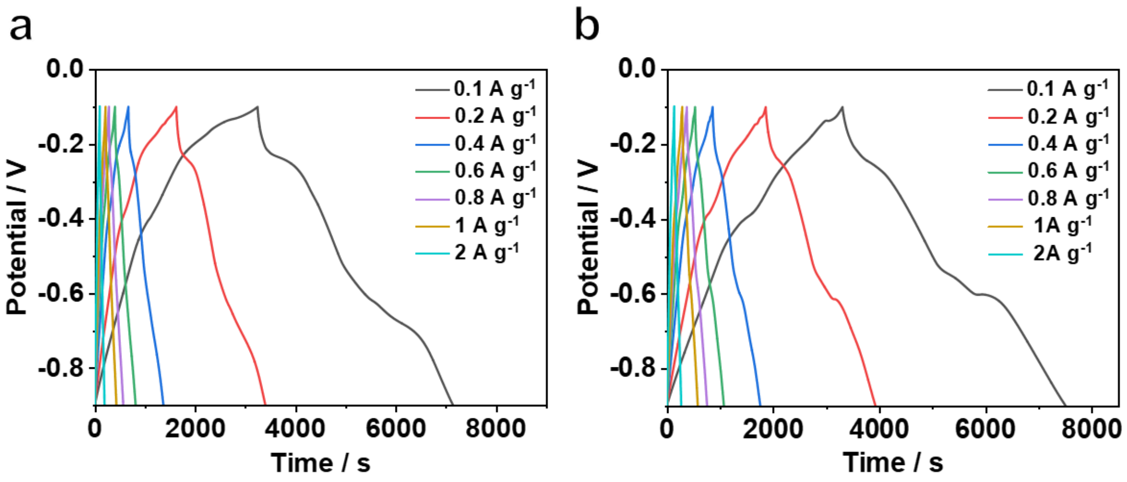

Figure S9. *GCD curves of (a) Cu-DO@GFF, (b) Cu-TO@GFF at 0.1-2 A g^{-1}.*

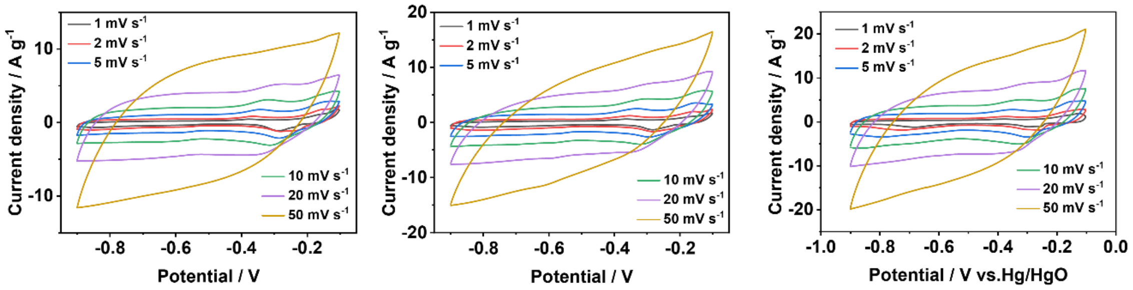

Figure S10. *CV curves of (a) Cu-DO@GFF, (b) Cu-TO@GFF and (c) Cu-MO@GFF at a scan rate range from 1 to 50 mV $^{s-1}$.*

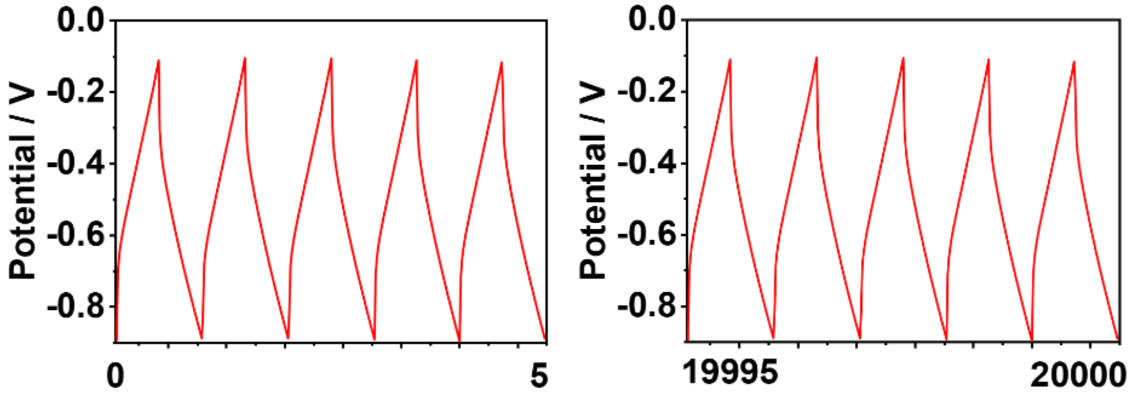

Figure S11. *The selected charge and discharge curves of Cu-MO@GFF from cycling test.*

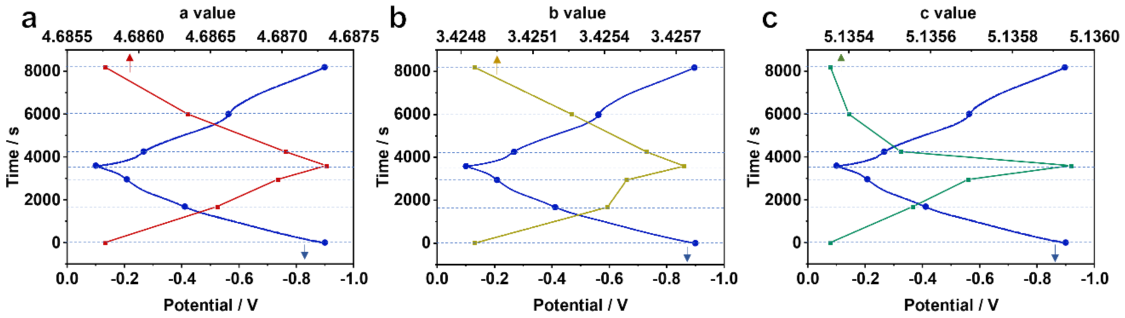

Figure S12. *Changes in the (a) a, (b) b, (c) c value of Cu-MO@GFF at various charge and discharge voltages.*

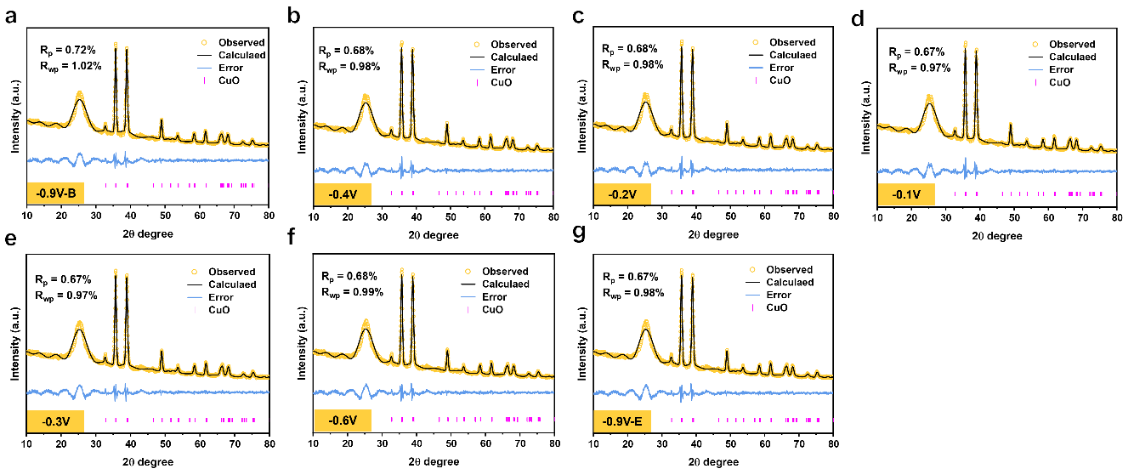

Figure S13. *Rietveld refinement analysis of XRD data of Cu-MO@GFF at (a) -0.9 V, (b) -0.4V, (c) -0.2V, (d) -0.1V, (e) -0.3V, (f) -0.6V and (g) -0.9 V.*

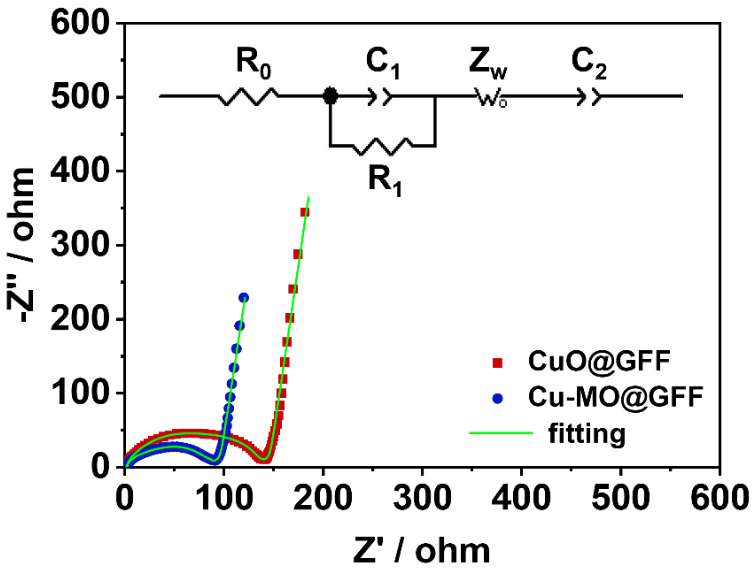

Figure S14. *EIS measurement of CuO@GF and Cu-MO@GFF. The inset is an equivalent circuit model.*

We compared the EIS results of CuO@GFF, Cu-MO@GFF and cycled Cu-MO@GFF, as shown in Figure S14, S20 and Table S3-4. From the fitting results, both raw CuO@GFF, Cu-MO@GFF, and cycled Cu-MO@GFF shows a similar R_0, with a value

of ~2 Ω, which is associated with the migration rate of ions in the electrolyte. However, a visible difference can be observed in the value of R_1, which are related to the charge transfer rate in electrode. Particularly, the CuO@GFF possesses a high R_1 value as 133.4 Ω, while it decreases to 83.4 Ω in Cu-MO@GFF. In addition, as the cycle prolongs, the Cu-MO@GFF still maintain a relatively low and stable R_1 value (Table S4). These impedance dynamics stability results verify that Cu-MO@GFF has good charge transfer ability through the multiply TM elements doping strategy.

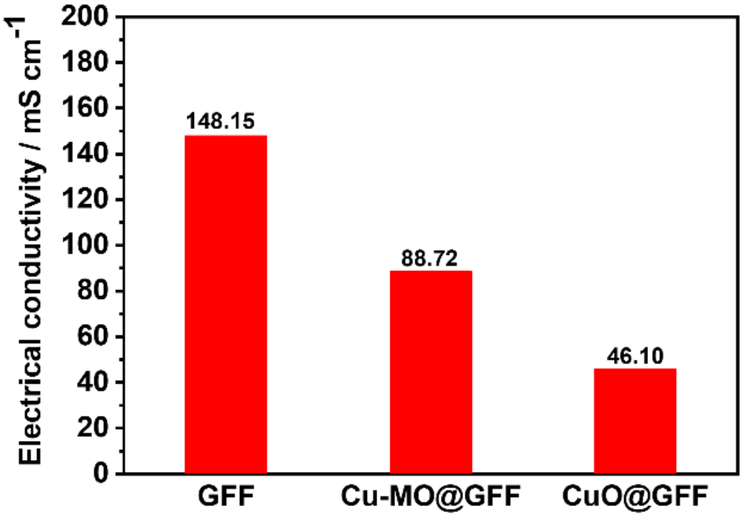

Figure S15. *Electronic conductivity of GFF, CuO@GFF, and Cu-MO@GFF.*

Due to a nature of lower electric conductivity of CuO, the introduction of CuO would significantly reduce the electronic conductivity of GFF to a low value (Figure S15). In a sharp contrast, the doping of multiple metal elements shows a positive function on improving the electronic conductivity of the copper oxide. Therefore, the multiple doping of transition metal elements would optimize the electron conductivity of oxide to enhance the electron conductivity of the composite fibers.

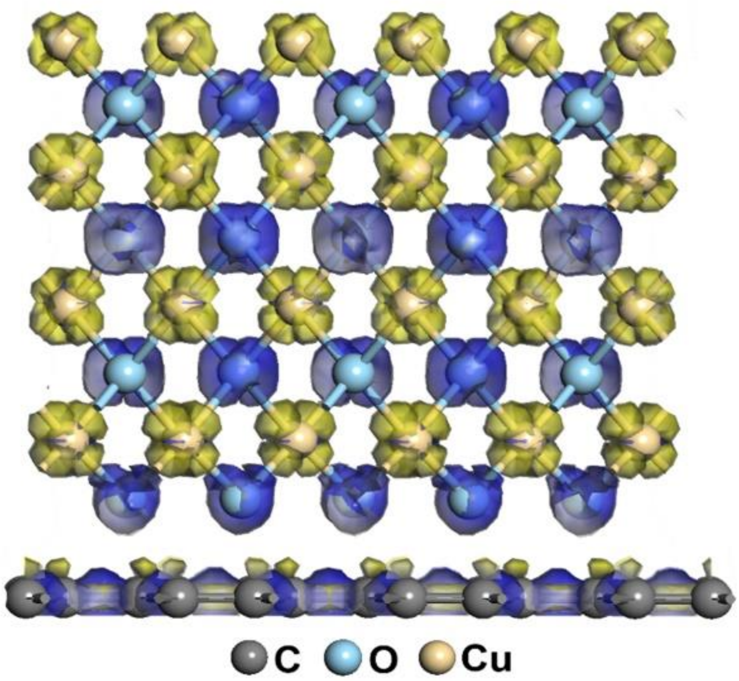

Figure S16. *The differential charge of CuO@GFF.*

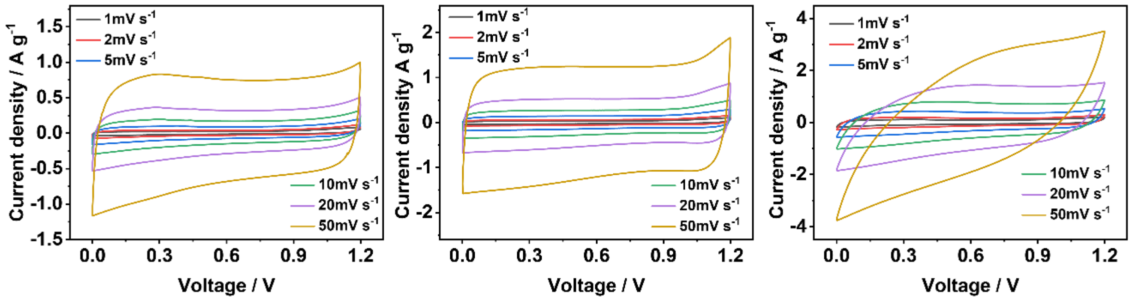

Figure S17. *CV curves of (a) GFF, (b) CuO@GFF and (c) Cu-MO@GFF at the scan rate of 1-50 mV* s^{-1}.

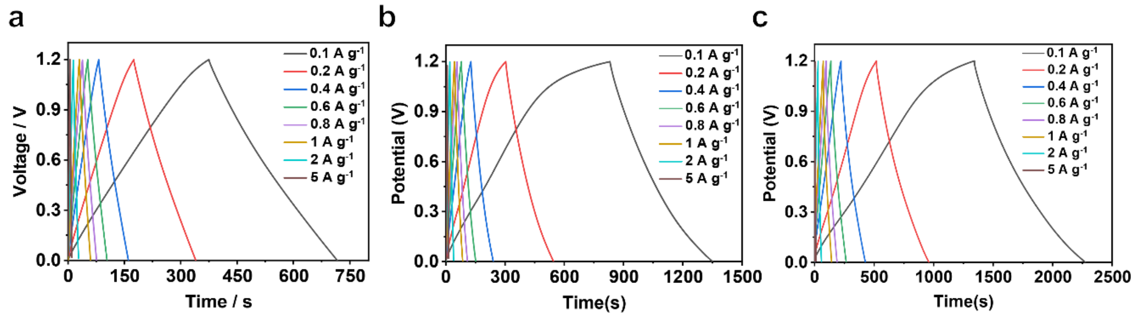

Figure S18. GCD curves of (a) GFF, (b) CuO@GFF and (c) Cu-MO@GFF FSC electrodes at 0.1-5 A g^{-1}.

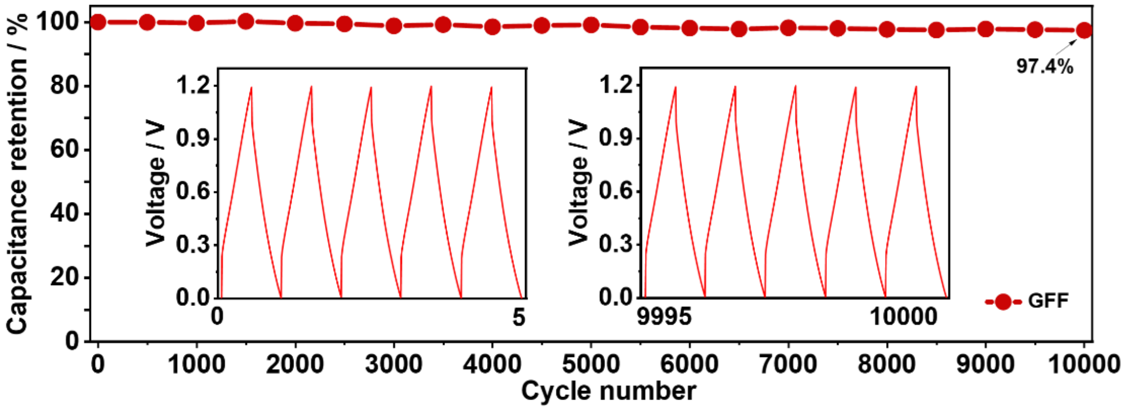

***Figure S19.** Cycling performance of GFF at 10 A g^{-1}*

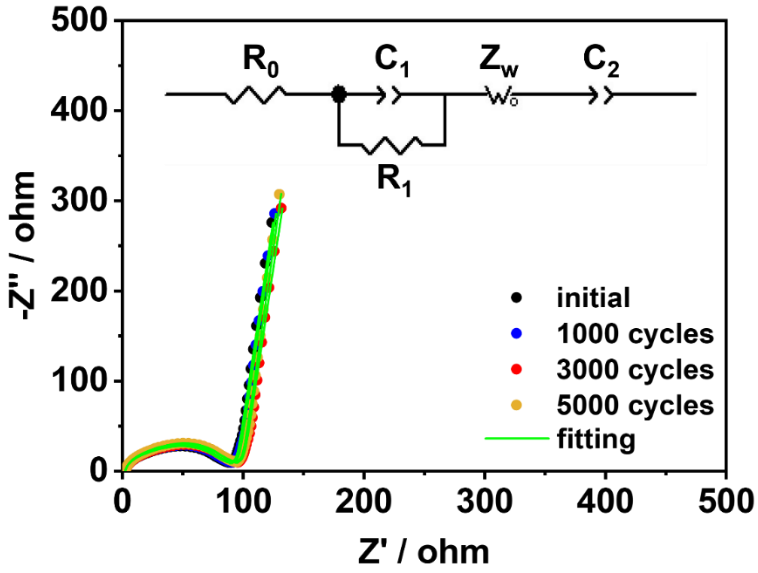

***Figure S20.** Kinetic analysis of the Cu-MO@GFF impedance, The inset is an equivalent circuit model.*

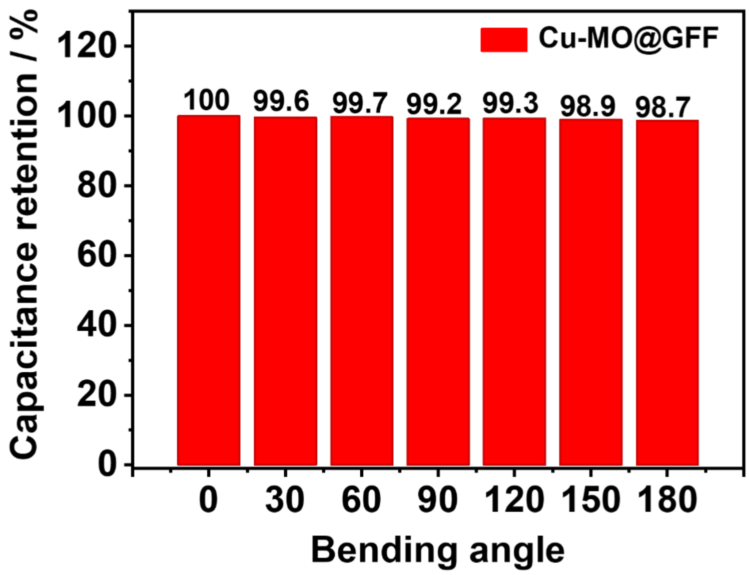

Figure S21. *Capacitance retention of Cu-MO@GFF FSC at different bending angles.*

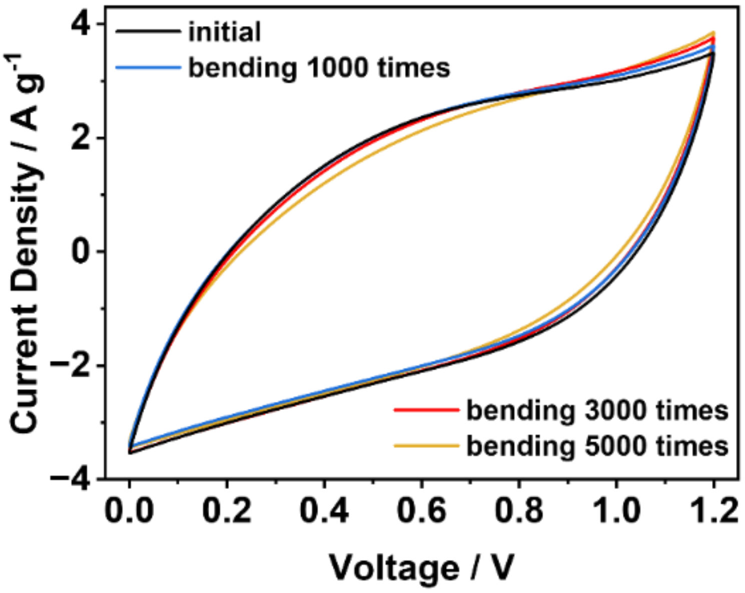

Figure S22. *CV curves of Cu-MO@GFF FSC after multiple bending at 60°.*

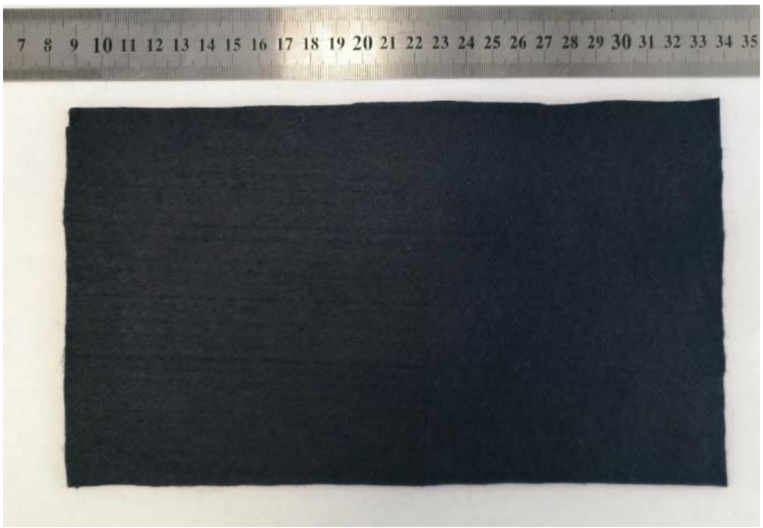

Figure S23. *Large-scale fabrication of Cu-MO@GFF.*

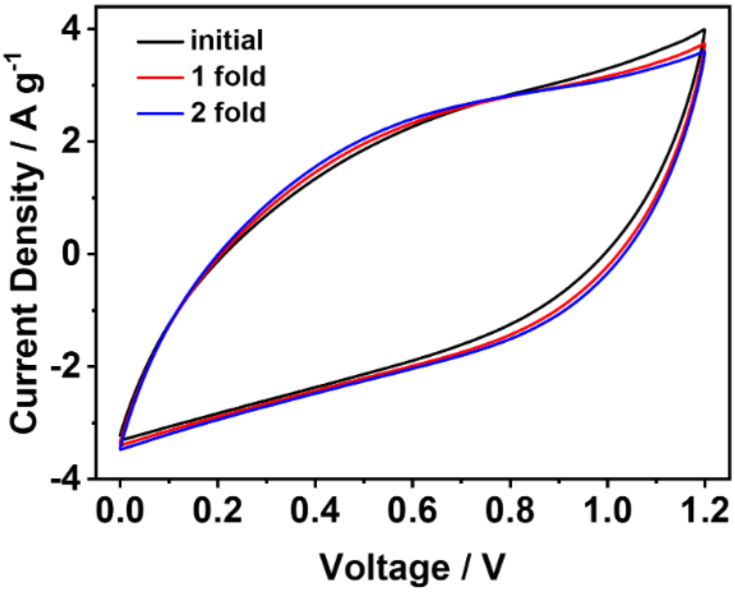

Figure S24. *CV curves of Cu-MO@GFF FSC after several folding times.*

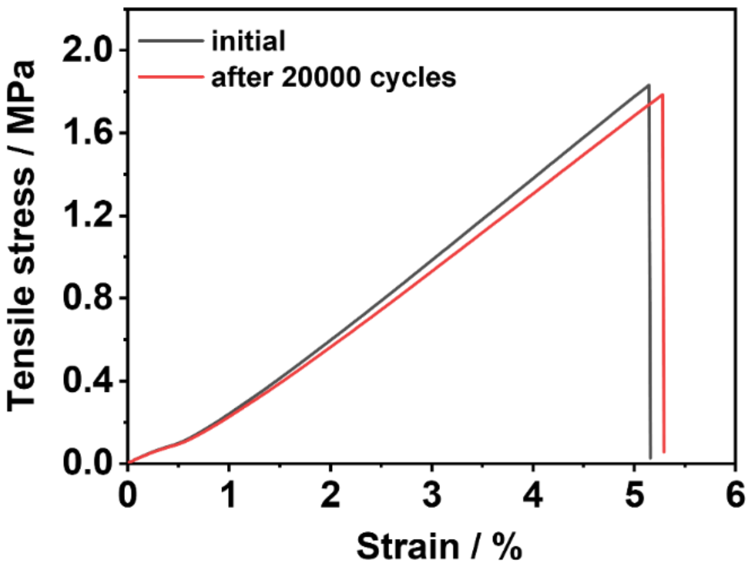

Figure S25. *Stress-strain curve of Cu-MO@GFF before and after cycling.*

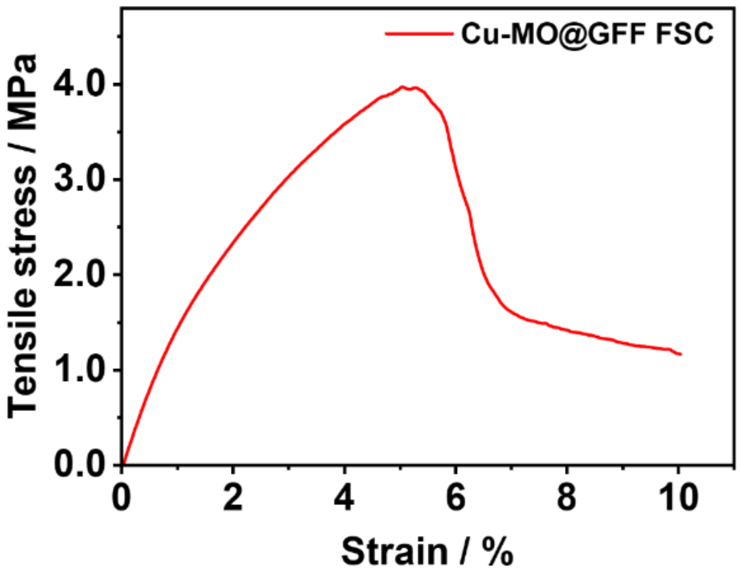

Figure S26. *Stress-strain curve of Cu-MO@GFF FSC.*

The stress-strain curves of Cu-MO@GFF before and after cycling are shown in Figure S25 to state the mechanical stability tests under repeated deformation. The fabric can still maintain a high fracture strength (1.831-1.786MPa) after long-term cycling, showing its high mechanical stability. Meanwhile, the mechanical strength of Cu-MO@GFF FSC is measured through the stress-strain curve, where it possesses a high

tensile strength (3.96MPa) and elongation (10.03%), ensuring the normal operation of FSC in various complex situations (Figure S26).

Electrode materials	electrolyte	Voltage window(V)	Capacitance ($F\ g^{-1}$)	Reference
HP-GFF	1M H_2SO_4	0-0.8	313	40
N_1SSC-700	6M KOH	0-1	325	41
BN-SAC-3	6M KOH	-1-0	304	42
TT(Zn/P)-700	6M KOH	-1-0.1	366	43
KAC-700	1M H_2SO_4	0-1	223	44
GMNAC	6M KOH	-0.2-0.8	310	45
BNMNC	3M KOH	-1-0	224	46
HPC	6M KOH	0-1	330	47
hp-NGR-1.0	6M KOH	-1-0	329	48
ON-CA	6M KOH	-1-0	385	49
CPIL	1M H_2SO_4	-0.2-0.8	359	50
B_1-SC	1M H_2SO_4	-0.7-0.3	296	51
Cu-MO@GFF	6M KOH	-0.9-(-0.1)	534	This work

Table S1. *The comparisons of electrode materials, electrolytes, working voltage and specific capacitance.*

Electrode	E_A (Wh kg^{-1})	P_A (W kg^{-1})	Capacitance Retention after 10,000 cycles	Electrolyte	Working Voltage (V)	Reference
RGO10E	5.56	496	84.2%	0.25M H_2SO_4	0-1	52
NMC-1	4.84	375	82.9%	3M KOH	0-0.8	53
PQLD-GO-10%	7.4	455	87.9%	3M Zinc trifluoromethanesulfonate	0-1	54
C@S-700A	3.458	650	87% after 5000 cycles	6M KOH	0-1.2	55
A-M/BC-0.2	9.63	250	83.46%	3M H_2SO_4	0-1	56
A-SC	10.17	165	85%	PE5/P(AA-co-AMPS)	0-0.8	57
NCNF2-900	5.7	1250	91% after 5000 cycles	PVA/KOH	0-0.8	58
Cu-MO@GFF	11.87	2287	89.3%	6M KOH	0-1.2	This work

Table S2. *Parallel comparisons in terms of electrode materials, energy density, power density, cycle performance, electrolytes and working voltage.*

Electrode	R_0/Ω	R_1/Ω
CuO@GFF	1.793	133.4
Cu-MO@GFF	2.056	83.4

Table S3. *R_0 and R_1 values for CuO@GFF and Cu-MO@GFF from the EIS fitting.*

Number of cycles	R_0/Ω	R_1/Ω
Initial	2.056	83.4
1000	2.089	85.26
3000	2.188	88.56
5000	2.136	88.64

Table S4. *R_0 and R_1 values for Cu-MO@GFF at various cycles from the EIS fitting.*

Supplementary Movie 1*: FSC powering yellow light*

Supplementary Movie 2*: FSC powering green light*

Supplementary Movie 3*: FSC powering wearable device*

Supplementary Movie 4*: FSC powering arm bracelet*
